# Exploring the Major Factors Affecting Generalized Anxiety Disorder in Korean Adolescents: Based on the 2021 Korea Youth Health Behavior Survey

**DOI:** 10.3390/ijerph19159384

**Published:** 2022-07-31

**Authors:** Mi-Lyang Kim, Kyulee Shin

**Affiliations:** 1 Department of Sport, Leisure, & Recreation, Soonchunhyang University, Asan-si 315358, Chungcheong-do, Korea; mlkim@sch.ac.kr; 2 Department of Sports Sciences, Seoul National University of Science & Technology, Seoul 01811, Korea

**Keywords:** generalized anxiety disorder, health behavior, Korea youth health behavior survey, Korean adolescents

## Abstract

(1) Background: Recently, the prevalence of generalized anxiety disorder (GAD) among adolescents has been higher than in adults. Early detection is important for treatment. Accordingly, although various factors affecting adolescents’ GAD have been studied, the body of research is fragmented, and an integrated analysis of the influencing factors is needed. Therefore, in this study, we intended to analyze various factors affecting GAD. (2) Methods: Using data from the Korea Youth Health Behavior Survey (2021), sociodemographic factors, negative emotion, and physical activity factors were selected. Correlation analysis, *t*-test, ANOVA, and multiple regression analysis were performed using SPSS 26.0. (3) Results: Perceived stress was found to be the factor that had the greatest influence on GAD. (4) Conclusions: The risk of GAD in Korean adolescents was found to increase in female students who had higher levels of perceived stress, and participated in less high-intensity or muscle-strengthening exercise.

## 1. Introduction

Generalized anxiety disorder (GAD) is a common mental disease that causes a range of considerable physical and mental symptoms [1]. It is characterized by excessive and widespread anxiety, as well as various physical symptoms such as muscle tension, poor concentration, and fatigue, according to the *DSM-5* of the American Psychiatric Association [2]. Currently, the most significant distinction between normal anxiety and GAD is the presence or absence of a triggering factor. Patients are diagnosed with GAD if they feel excessively anxious despite the absence of factors or events that may cause anxiety, and if symptoms accompanied by physical symptoms persist for more than 6 months and interfere with daily life [1].

According to the Korea Health Insurance Review and Assessment Service (2021), the number of patients receiving treatment for GAD is increasing every year, from 75,127 in 2016 to 83,195 in 2020 [3]. The lifetime prevalence rate is relatively high too, with about 5% of Koreans experiencing GAD. However, due to the presence of more ambiguous symptoms than other types of anxiety disorders, it is difficult to distinguish the symptoms of GAD from those of regular anxiety. Furthermore, because some people do not recognize their condition as a disease, it is less likely to be detected early or labeled as problematic behavior [4]. As a result, if proper treatment is not received, GAD can become chronic, leading to other types of mental disorders such as social phobia, panic disorder, and depression.

According to related data from the Korea National Health Insurance Corporation (2019), by age of patients treated for anxiety disorders (including GAD, panic disorder, obsessive-compulsive disorder, and post-traumatic stress disorder), the rate of increase was highest among those in their 20s (86.2%), followed by teenagers (46.5%) and those in their 30s (45.9%) [5]. In addition, Lim reported that one out of nine Korean adolescents (about 11.2%) belonged to the high-risk group for GAD, showing that the prevalence of GAD in adolescence is much higher than in adults [6].

According to the psychosocial developmental theory of Erickson, an American psychoanalyst, adolescence between the ages of 12 and 20 years represents a transition from childhood to adulthood, an important time to explore the self, contemplate future-related conflicts, and form identity. Erikson considered the process of coping with and adapting to the external environment very important at each development stage, and the prolonged COVID-19 pandemic has jeopardized this process among adolescents. It can be argued that not only was students’ learning anxiety amplified in a situation where normal school attendance was difficult, but also mental health problems, including GAD, became more frequent due to problems such as social isolation and the neglect of parenting acting in complex interactions [7]. Taking the impact of COVID-19 into account, this is a crucial time to raise social interest in the problem of GAD in adolescence and prepare appropriate preventive measures.

According to earlier research [8,9,10], GAD in adolescence is connected with psychosocial issues, educational challenges, and poor self-esteem, and can evolve into a chronic handicap without effective treatment and with time. At this point, the intensity of the symptoms tends to fluctuate over time, and numerous stresses are the primary aggravators of the symptoms of GAD. Specifically, age and chronic illness have been shown to be unfavorable predictors of GAD [10]. However, adolescents with GAD have difficulties identifying anxiety disorders owing to the absence of observable behavioral changes, despite the fact that adaption issues and other forms of illnesses are associated with maturity [11]. Therefore, it is vital to thoroughly diagnose GAD early to determine the most effective treatment option. In addition, it is essential to identify the risk group in advance by determining the numerous elements that contribute to GAD in adolescents. In this research, we thus focused on Korean middle school pupils and sought to determine the proportionate effect of several variables on their GAD.

While most research on GAD in Korea has primarily focused on the diagnosis, evaluation, and specific treatment of adults, some research has focused on the prevalence of GAD in adolescents [1,12,13]. In these studies, sex, age, grade, chronic disease, social class, and sociopsychological factors were commonly viewed as factors influencing GAD [12]. In particular, Lim (2021) analyzed the factors related to GAD in Korean adolescents, and found that the prevalence of GAD was linked to health-related behavioral factors such as drinking and smoking, subjective perception of health or stress, and feelings of hopelessness and depression [6]. The classification and examination of the same characteristic psychological factors were performed in the present study.

In addition to the sociodemographic and psychosocial factors identified in the preceding study, it has been discovered that factors such as the level of physical activity, distortion of body image, and subjective body perception have a significant impact on the mental health of adolescents [14,15]. However, previous studies that revealed the factors influencing adolescent GAD were limited by their analysis of the specific factors on which each researcher focused, resulting in fragmented results.

Therefore, the purpose of this study was to perform a multiple regression analysis to determine the factors affecting GAD in Korean adolescents using large-scale national statistics, thus revealing the relative influence of each factor on the GAD of Korean adolescents.

## 2. Materials and Methods

### 2.1. Research Subjects and Data

Statistical data from the 2021 survey of the 17th Youth Health Behavior Survey were downloaded and utilized in accordance with the disclosure and management regulations for raw data to comprehend the health behavior of Korean adolescents (the following URL provides the questionnaires and raw data: https://www.kdca.go.kr/yhs/home.jsp (accessed on 8 July 2022)). This survey’s population consisted of middle and high school students in Korea as of April 2021, and the sampling procedure included stratification, distribution, and sampling stages. The population was stratified into 117 layers, and after classifying 17 cities and counties into large, medium, and small cities and counties; it was classified into 39 areas based on geographical accessibility, the number of schools, population, living environment, smoking rate, and drinking rate. We chose 400 middle schools and 400 high schools during the sample distribution phase. Using the stratified colony sampling method, the school was selected as the primary sampling unit, and one class from each grade was randomly selected from the sample school. All students in the sampled class were surveyed. A total of 59,426 students, excluding long-term absentees, disabled children, and students with text-deciphering difficulties, were surveyed, and 54,848 students from 796 schools participated in the survey with a response rate of 92.9 percent. Table 1 lists the subjects’ general characteristics.

### 2.2. Research Variables

The variables in this study were selected through expert advice and review of previous studies. Sex, school year, academic performance, and economic status were selected as socio-demographic variables. Perceived stress, sadness, suicidal thoughts, and loneliness were selected as predictor factors affecting GAD. In order to determine the effect of exercise on GAD, physical activity, high-intensity exercise, and muscle-strengthening exercise were selected as variables.

In the analysis, academic performance, economic status, and perceived stress were reverse-coded. The sex variable was transformed to a dummy variable for the analysis. The economic condition was categorized according to the subject’s response as upper, mid-upper, middle, mid-lower, or lower on a 5-point scale.

The generalized anxiety disorder scale consists of a total of 7 items, and each item is rated from 0 to 4 points, for a total score of 21 points. A score of 0–4 is considered minimal GAD, 5–9 mild GAD, 10–14 moderate GAD, and 15–21 severe GAD. The higher the score, the higher the GAD level. The reliability of the scale was 0.89.

### 2.3. Analysis Method

The following analysis method was applied using SPSS 26.0 (New York, NY, USA) for the purposes of this investigation. First, frequency analysis and descriptive statistics analysis were performed to understand the general characteristics of the research subjects. Second, correlation analysis was performed to understand the relationship between variables; third, multiple regression was performed to understand the relative size of the factors affecting GAD.

## 3. Results

### 3.1. Correlation among Variables

The results from examining the relationship between GAD and sociodemographic variables were as follows: sex (r = 0.164 ***, *p* < 0.001), school year (r = 0.036 ***, *p* < 0.001), perceived stress (r = 0.544 ***, *p* < 0.001), sadness (r = 0.443 ***, *p* < 0.001), suicidal thoughts (r = 0.407 ***, *p* < 0.001), and loneliness (r = 0.531 ***, *p* < 0.001) showed significant positive correlations; academic performance (r = −0.066, *p* < 0.001), economic status (r = −0.099 ***, *p* < 0.001), physical activity (r = −0.041 ***, *p* < 0.001), high-intensity exercise (r = −0.058 ***, *p* < 0.001), and muscle-strengthening exercise (r = −0.072 ***, *p* < 0.001) showed significant negative correlations (Table 2).

### 3.2. Differences in GAD by Sex and School

To examine GAD differences between boys and girls and between middle and high school students, independent sample *t*-tests were conducted. The test results are summarized in Table 3. The result of the independent sample *t*-test of GAD according to sex was *t* = 38.881 ***, *p* = 0.000. It was found to be statistically significant based on the significance level of 0.05, so we found a difference in the prevalence of GAD depending on sex. Female students (1.699 ± 0.673) responded with higher GAD scores than male students (1.488 ± 0.595).

The independent sample *t*-test result on whether there was a difference in the prevalence of GAD between middle and high school students was *t* = −4.852, ***, *p* = 0.000, which was statistically significant based on the significance level of 0.05, so we found that the prevalence of GAD was different between middle and high school students. Additionally, high school students (1.604 ± 0.653) responded with higher GAD scores than middle school students (1.578 ± 0.633).

### 3.3. Differences in GAD by Acadimic Performance and Economic Status

ANOVA was conducted to determine whether there were differences in GAD according to five categories of academic performance. The ANOVA results showed a significant difference in the prevalence of GAD depending on academic performance (F = 91.887, ***, *p* < 0.001) (Table 4). The Scheffe test was performed as a post hoc test, and the results were as follows: the GAD of lower-performing students (1.72) was higher than that in other categories (1 > 2, 3, 4, 5 ***), and the GAD of ‘mid-lower’ (1.63) was higher than GAD of 3, 4, and 5 (2 > 3, 4, 5 ***). There was no difference in GAD between middle, mid-upper, and upper.

The difference in GAD according to the five categories of economic status was also examined by ANOVA, and the results were significantly different (F = 192.856, ***, *p* < 0.001) (Table 5). The Scheffe test was performed as a post hoc test, and the results were as follows: GAD of ‘lower’ (1.90) was higher than that in other categories (1 > 2, 3, 4, 5 ***), GAD of ‘mid-lower’ (1.76) was higher than that of 3, 4, 5 (2 > 3, 4, ***), and GAD of ‘middle’ and ‘mid-upper’ was higher than GAD of ‘upper’. There was no difference in GAD between middle and mid-upper economic status.

### 3.4. Impact of Major Factors on GAD

Multiple regression analysis was conducted to see if the eleven factors significantly predicted GAD. Table 6 shows that the model explained a significant amount of the variance in GAD prevalence (F = 3998.212, ***, *p* < 0.001, R^2^ = 0.445, R^2^ adjusted = 0.445). The Durbin-Watson statistic was 1.958, confirming that there was no autocorrelation of the dependent variable. Because none of the VIFs exceeded 10 and the tolerance limit was 0.487 to 0.947, which is greater than 0.1, there was no strong collinearity in this regression. 

As shown in Table 6, the results of multiple regression analyses show that sex (*β* = 0.029, *p* < 0.001), economic status (*β* = −0.017, *p* < 0.001), perceived stress (*β* = 0.305, *p* < 0.001), sadness (*β* = 0.156, *p* < 0.001), suicidal thoughts (*β* = *0*.151, *p* < 0.001), loneliness (*β* = 0.264, *p* < 0.001), physical activity (*β* = 0.010, *p* < 0.05), high-intensity exercise (*β* = −0.017, *p* < 0.001), and muscle-strengthening exercise (*β* = −0.019, *p* < 0.001) significantly affected GAD, while school year (*β* = −0.002, *p* > 0.05) and academic performance (*β* = −0.002, *p* > 0.05) were found to have no significant effect on GAD.

## 4. Discussion

A current problem in Korean society is the cramming education system and intense competition for university admission. According to a related study, British adolescents, who are known to have relatively high educational commitment among European countries, study for 6.9 h/day, far less than the 11.5 h of Korean students [16]. Additionally, according to the OECD’s Program for International Student Assessment (PISA), the academic achievement scores of Korean youths are consistently higher than the world average every school year [17]. In Korea’s competitive social structure, where the academic clique is recognized as one of the important attainments, the middle and high school period holds great importance for gaining an edge in the university admission competition.

Adolescence is a time when emotional and intellectual interactions form a more complex structure than in childhood, and underestimation or overestimation of the self are opposed to each other. For this reason, neuroticism and mental confusion are often experienced in adolescence [18]. These developmental characteristics accompany the stress of survival in a competitive system, so the middle and high school period in Korea presents a very high probability of developing GAD, overanxiety, and anxiety symptoms. Therefore, in the present study, we focused on the factors influencing GAD in Korean middle and high school students to provide basic data for the development of a national intervention program for GAD. This is necessitated by the difficulty of mediating all the factors that influence GAD, as well as the need to focus on more than a single influencing factor.

Therefore, in this study, using the 17th Korea Youth Risk Behavior Survey (2021) of the Korea Disease Control and Prevention Agency (KDCA), sociodemographic factors, the negative emotions of middle and high school students, and physical activity were simultaneously considered. The following are the results of major factors affecting GAD in Korean middle and high school students.

First, the sociodemographic factors, such as sex, school year, academic performance, and economic status were found to influence GAD. As a result of analyzing the difference in GAD according to sex through an independent *t*-test, the prevalence of GAD in female students was significantly higher than that in male students. In other words, the risk of GAD was higher in female students than in male students. Avenevoli et al.’s (2013) GAD study of adolescents in the United States also revealed this sex disparity, discovering that the ratio of female students was 55–60% [19]. Lim (2021), who analyzed the factors influencing GAD among Korean adolescents, also found that female students had a 1.24 times higher prevalence of GAD than male students, supporting the results of this study [6]. According to the results of the independent *t*-test for comparing GAD according to middle and high school students, the prevalence of GAD in high school students was higher than that in middle school students. The results of the ANOVA, which tested the differences in GAD for five categories of academic performance and economic status, showed that academic performance and economic status affect the GAD of middle and high school students. In studies by Kessler et al. (2005) and Lim (2021), the prevalence of GAD was 1.09 times higher in the lower economic class than in the upper class [6,20]. Therefore, sex, school year, academic performance, and economic status were included as factors affecting the degree of GAD [6,21].

Second, multiple regression analysis was conducted to identify which factors significantly affect GAD and which factors have the greatest influence. The results of multiple regression analysis shows that sex, economic status, perceived stress, sadness, suicidal thoughts, loneliness, physical activity, high-intensity exercise, and muscle-strengthening exercise significantly affected GAD, while school year and academic performance were found to have no significant effect on GAD. Perceived stress was found to be the factor that had the greatest influence on GAD, followed by loneliness, sadness, and suicidal thoughts. 

Stress-related GADs are often described by the stress-diathesis model. This theory states that GAD is triggered when various stresses are applied to people who are born with or acquired vulnerabilities related to major mental disorders. Stress dealt with in anxiety disorders is called psychological trauma, and exposure to such psychological trauma is known to increase the risk of anxiety disorders such as GAD [22], which is in line with the results of this study.

Third, high-intensity activity and muscle-strengthening exercise showed an inverse correlation with GAD. The reality of competitive education in Korea is that it is difficult for middle and high school students to perform enough exercise [17]. According to the World Health Organization’s (2019) Global Recommendation on Physical Activity for Health, 94.2% of Korean adolescents lack exercise, ranking first among 146 countries in the world [23]. In particular, 97.2% of female students were judged to lack exercise, indicating that most Korean adolescents do not engage in sufficient physical activity to maintain and develop their physical or mental health.

The World Health Organization (2020) recommends at least 60 min of moderate-intensity physical activity and muscle-strengthening exercise per day on average for children and adolescents (5–17 years) [23]. Appropriate physical activity is reported to reduce the occurrence of health-harmful factors in adults and old age, which supports the results of this study. Therefore, it is necessary to prepare practical support measures to help Korean middle and high school students recognize the importance of appropriate physical activity, who have a sedentary lifestyle due to the prioritization of education. However, as the explanatory power of physical activity factors for GAD in middle and high school students was found to be very insignificant, at about 1%, further research is needed to determine whether this is due to specific Korean circumstances such as excessive competition for entrance exams.

Combining the above results, we suggest that middle and high school students from a lower economic level and with a higher level of perceived stress are more likely to develop GAD, with the added layer of a sex disparity that places female students at higher risk. The major variables identified as risk factors for GAD in Korean middle and high school students through this study can be used to develop policy priorities for early detection, prevention, and appropriate treatment of GAD in Korean middle and high school students.

## 5. Conclusions

This study was conducted to comprehensively understand the effects of sociodemographic, negative emotion, and physical activity factors on GAD in Korean middle and high school students using the Korea Youth Health Behavior Survey (2021).

First, we found that sociodemographic factors (sex, school year, academic performance, and economic status) affect GAD.

Second, the results of the independent *t*-test analysis showed that GAD according to sex and middle school and high school students showed a significant difference.

Third, the results of the ANOVA analysis of GAD according to the five categories of academic performance and ecological status showed a significant difference in GAD depending on academic performance and economic status.

Fourth, multiple regression analysis was conducted to identify which factors significantly affect GAD and which predictors have the greatest influence. The results of multiple regression analysis showed that sex, economic status, perceived stress, sadness, suicidal thoughts, loneliness, physical activity, high-intensity exercise, and muscle-strengthening exercise significantly affected GAD, while school year and academic performance were found to have no significant effect on GAD. Perceived stress was found to be the predictor that had the greatest influence on GAD, followed by loneliness, sadness, and suicidal thoughts.

Fifth, although the explanatory power of the three physical activity factors was not high, all of them were found to have a significant effect on GAD. High-intensity activity and muscle-strengthening exercise showed an inverse correlation with GAD, and high-intensity exercise and muscle-strengthening exercise were more effective in reducing GAD than routine physical activity.

Therefore, we concluded that the risk of GAD in middle and high school students increases when the level of perceived stress is high, and high-intensity exercise and muscle-strengthening exercise were the most effective in reducing GAD.

Because this is a study of Korean middle school students, it may be difficult to generalize the findings to other countries. However, this study makes an important contribution in that we revealed the most influential variables affecting GAD by comprehensively analyzing the factors affecting GAD. The primary objective of this study was to identify the most influential variables among those known to influence GAD in middle and high school students. The purpose of this research was to establish evidence that can distinguish risk groups for generalized anxiety disorder in middle school students. It should not be overlooked, however, that these studies utilizing national statistics can analyze data from multiple perspectives.

## Figures and Tables

**Table 1:** Analysis of the characteristics of participants.

Variables		N	%
Sex	Male	28,401	51.8
	Female	26,447	48.2
School year	Middle school	24,833	45.3
	High school	30,015	54.7
Academic performance	Upper	7084	12.9
	Mid-upper	13,444	24.5
	Middle	16,903	30.8
	Mid-lower	12,004	21.9
	Lower	5413	9.9
Economic status	Upper	5944	10.8
	Mid-upper	15,624	28.5
	Middle	27,077	49.4
	Mid-lower	5091	9.3
	Lower	1112	2.0
Total		54,848	100

**Table 2:** Correlations among the study variables.

	1	2	3	4	5	6	7	8	9	10	11	12
1	1											
2	0.005	1										
3	−0.007 *	−0.141 ***	1									
4	−0.042 ***	−0.129 ***	0.305 ***	1								
5	0.162 ***	0.066 ***	−0.067 ***	−0.092 ***	1							
6	0.106 ***	0.030 ***	−0.084 ***	−0.055 ***	0.377 ***	1						
7	0.106 ***	−0.013 **	−0.054 ***	−0.072 ***	0.326 ***	0.389 ***	1					
8	0.165 ***	0.038 ***	−0.063 ***	−0.177 ***	0.465 ***	0.418 ***	0.345 ***	1				
9	−0.241 ***	−0.120 ***	0.033 ***	0.060 ***	−0.059 ***	0.019 ***	−0.012 **	−0.022 ***	1			
10	−0.290 ***	−0.139 ***	0.023 ***	0.069 ***	−0.066 ***	0.013 **	−0.010 *	−0.031 ***	0.655 ***	1		
11	−0.348 ***	−0.056 ***	−0.011 **	0.049 ***	−0.080 ***	0.006	−0.022 ***	−0.042 ***	0.502 ***	0.566 ***	1	
12	0.164 ***	0.036 ***	−0.066 ***	−0.099 ***	0.544 ***	0.443 ***	0.407 ***	0.531 ***	−0.041 ***	−0.058 ***	−0.072 ***	1

* *p* < 0.05, ** *p* < 0.01, *** *p* < 0.001, 1. Sex. 2. School year. 3. Academic performance. 4. Economic status. 5. Perceived stress. 6. Sadness. 7. Suicidal thoughts. 8. Loneliness. 9. Physical activity. 10. High-intensity exercise. 11. Muscle-strengthening exercise. 12. GAD.

**Table 3:** Comparison of mean GAD scores by sex and school.

Variables		N	Mean	SD	*t*	*p*
Sex	Male	28,401	1.488	0.595	38.881 ***	0.000
	Female	26,447	1.699	0.673		
School	Middle school	30,015	1.578	0.633	−4.852 ***	0.000
	High school	24,833	1.604	0.653		

*** *p* < 0.001.

**Table 4:** Comparison of mean GAD scores by academic performance.

Academic Performance		N	Mean	SD	F	Post Hoc
1	Lower	5413	1.72	0.77	91.887 ***	1 > 2 ***
2	Mid-lower	12,004	1.63	0.65		1 > 3, 4, 5 ***
3	Middle	16,903	1.55	0.61		2 > 3, 4, 5 ***
4	Mid-upper	13,444	1.57	0.61		
5	Upper	7084	1.55	0.64		
Total		54,848	1.59	0.64		

*** *p* < 0.001.

**Table 5:** Comparison of mean GAD scores by economic status.

Economic Status		N	Mean	SD	F	Post Hoc
1	Lower	1112	1.90	0.88	192.856 ***	1 > 2, 3, 4, 5 ***
2	Mid-lower	5091	1.76	0.71		2 > 3, 4, 5 ***
3	Middle	27,077	1.58	0.62		3, 4 > 5 ***
4	Mid-upper	15,624	1.56	0.61		
5	Upper	5944	1.51	0.65		
Total		54,848	1.59	0.64		

*** *p* < 0.001.

**Table 6:** Impact of major factors on GAD.

Predictor	B	SE	*β*	*t*	*p*	Tolerance	VIF
Constant	−0.034	0.015		−2.244 *	0.025		
Sex	0.037	0.004	0.029	8.212 ***	0.000	0.835	1.198
School year	−0.001	0.001	−0.002	−0.633	0.527	0.947	1.056
Academic performance	−0.001	0.002	−0.002	−0.734	0.463	0.890	1.123
Economic status	−0.013	0.002	−0.017	−5.042 ***	0.000	0.886	1.129
Perceived stress	0.204	0.003	0.305	81.296 ***	0.000	0.718	1.393
Sadness	0.226	0.005	0.156	41.738 ***	0.000	0.728	1.374
Suicidal thoughts	0.291	0.007	0.151	42.141 ***	0.000	0.789	1.268
Loneliness	0.159	0.002	0.264	68.852 ***	0.000	0.689	1.452
Physical activity	0.003	0.001	0.010	2.363 *	0.018	0.543	1.842
High-intensity exercise	−0.006	0.002	−0.017	−3.752 ***	0.000	0.487	2.054
Muscle-strengthening exercise	−0.007	0.002	−0.019	−4.651 ***	0.000	0.615	1.626

*** *p* < 0.001, * *p* < 0.05, R^2^ = 0.445, R^2^ adjusted = 0.445, F = 3998.212 ***, Durbin-Watson d = 1.95.

## Data Availability

Data are available upon request at https://www.kdca.go.kr/yhs/ (accessed date 1 February 2022).
